# LONGITUDINAL AUTOREGRESSIVE AND CROSS-LAGGED EFFECTS BETWEEN FRAILTY AND COGNITIVE FUNCTION OVER 14 YEARS

**DOI:** 10.1093/geroni/igae098.4018

**Published:** 2024-12-31

**Authors:** Min Jung Kim, Chang Gi Park

**Affiliations:** Gachon University, Incheon, Republic of Korea; University of Illinois Chicago, Chicago, Illinois, United States

## Abstract

Frailty and cognitive impairment are common geriatric conditions with a strong association, though it remains unclear whether frailty leads to cognitive impairment or vice versa. Both conditions are typically irreversible and follow worsening trajectories that vary among individuals. This study examined the longitudinal autoregressive and reciprocal causal relationships between frailty and cognitive impairment, accounting for within-person processes, using a nationally representative sample, Korean Longitudinal Study of Aging 2006 to 2020. Frailty Index (FI) scores were derived from 33 items, while cognitive impairment was assessed using the Korean Mini-Mental Status Examination (K-MMSE). The sample included 3,853 adults aged 45 years or older at baseline with complete FI and K-MMSE data. A random-intercept cross-lagged panel model with robust maximum likelihood estimation was employed to address non-normality in the outcomes. The analysis revealed significant reciprocal causal relationships between FI and K-MMSE scores, with the strength of these relationships gradually increasing over time. The magnitudes of these relationships were comparable at each time point. Both FI and K-MMSE exhibited significant autoregressive effects, with FI showing consistently higher autoregressive coefficients than K-MMSE over the 14-year period. These findings suggest that frailty and cognitive function may serve as therapeutic targets for each other, with frailty being more persistent over time and cognitive impairment potentially influenced by other factors, including changes in frailty. Early interventions targeting either condition may yield synergistic preventive effects on both frailty and cognitive impairment, offering a valuable approach to managing these debilitating conditions.

